# Dietary Patterns Are Associated With Multi-Dimensional Cognitive Functions Among Adults Aged 55 and Older in China

**DOI:** 10.3389/fnut.2022.806871

**Published:** 2022-02-17

**Authors:** Qiumin Huang, Hongru Jiang, Jiguo Zhang, Xiaofang Jia, Feifei Huang, Huijun Wang, Bing Zhang, Liusen Wang, Minxia Gu, Yuelong Huang, Wei Shi, Yuxia Ma, Xinjing Zhang, Zhihong Wang

**Affiliations:** ^1^National Institute for Nutrition and Health, Chinese Center for Disease Control and Prevention, Beijing, China; ^2^Ninghai Country Center for Disease Control and Prevention, Ningbo, China; ^3^Hunan Provincial Center for Disease Control and Prevention, Changsha, China; ^4^Shaanxi Provincial Center for Disease Control and Prevention, Xian, China; ^5^School of Public Health, Hebei Medical University, Shijiazhuang, China

**Keywords:** dietary patterns, cognition, cognitive domains, mild cognitive impairment, Chinese population

## Abstract

**Background:**

The intake of certain food and nutrients may play a crucial role in cognitive health. However, research on the relationship between dietary patterns and cognitive function is limited. This study aims to investigate the associations between dietary patterns and multi-dimensional cognitive functions, such as global cognitive status and related domain profiles, mild cognitive impairment (MCI), and four major subtypes of Chinese adults.

**Methods:**

Using the baseline data from the Community-based Cohort Study on Nervous System Diseases (2018–2019), we selected 4,309 Chinese adults aged 55 years and older as subjects with complete diet, cognition, and other related data. We collected food data for the past 12 months with a valid semi-quantitative food frequency questionnaire. Diving 49 food items into 13 subgroups, we used factor analysis to derive the main dietary patterns. We evaluated cognitive functions based on the scores of the Montreal Cognitive Assessment (MoCA) and used quantile regression and multivariable logistic regression to examine the relationship between dietary patterns and cognitive-related outcomes.

**Results:**

We identified four dietary patterns, explaining 50.11% of the total variance: “meat-preferred” pattern, “plant-preferred” pattern, “eggs- and dairy-preferred” pattern, and “grain-preferred” pattern. After adjusting for all potential confounders, the “meat-preferred” pattern and the “plant-preferred” pattern were associated with higher scores of global cognition and several cognitive domains (*p* <0.05), while the “grain-preferred” pattern was associated with lower scores of global cognition (β = −0.36, *p* <0.05), execution (β = −0.19, *p* <0.05), visuospatial (β = −0.09, *p* <0.05), and language (β = −0.05, *p* <0.05). Adults adhering to the “meat-preferred” pattern and the “plant-preferred” pattern had decreased odds of MCI and some MCI subtypes (*p-trend* <0.05); in contrast, those in the top quartiles of the “grain-preferred” pattern had increased odds of MCI [adjusted odds ratio (*AOR*) = 1.34, 95% CI: 1.11–1.63, *p-trend* = 0.003].

**Conclusions:**

Adhering to the “plant-preferred” pattern and the “meat-preferred” pattern may help improve the multi-dimensional cognitive functions; on the contrary, adhering to the “grain-preferred” pattern may worse cognitive health. More prospective studies in this field are needed to strengthen the evidence.

## Introduction

There are 264.02 million people aged 60 years or older in China, accounting for 18.7% of the total population of 1.40 billion in 2020, an increase of about 5.4% over 2010. Due to the aging population, neurodegenerative disorders are expected to increase significantly. In 2020, it was reported that an estimated 9.83 million Chinese aged 60 years and older suffered from Alzheimer's disease (AD), whose cognition declines with age; and 38.77 million suffered from mild cognitive impairment (MCI), the most common outcome of pathological cognitive decline in the elderly (1). MCI, as an intermediate state, 8.1% of MCI may become AD (2) and 10–14% of MCI may return to normal cognition (3). To date, there are no effective measures to fight the progression of AD (4). Therefore, it is extremely important to fully investigate relevant risk factors associated with AD and provide sight into evidence-based intervention for early prevention among adults at early stages.

Diet may have an impact on the cognitive health. Studies in the United States, France, Brazil, and China found that regular consumption of fish, nuts, fruit, or vegetables is associated with improved cognitive function (5–7). Considering the complex interaction among various nutrients and food, research on the relationship between dietary patterns and cognition has received more and more attention. Observation studies showed that adherence to the Mediterranean diet can improve the global cognitive function (8) and decrease the risk of MCI in the elderly (9). Among the elderly in Japan, the “plant food and fish” pattern with high intake of vegetables, soy products, fruit, and fish determined by factor analysis is positively correlated with cognitive function score (10). Almost all of these studies have been carried out in Western countries, some of which have limitations in controlling cofounding factors. However, large-scale research in China is limited, and few studies have explored the associations between dietary patterns and cognitive outcomes in the subtypes of MCI. Accumulating more evidence in different regions of China is crucial to prevent the cognitive decline. Because of food items, nutrients in foods, and eating behaviors, especially the obvious nutrition transition in China. The beneficial dietary patterns identified in some regions may not be consistent with those in other regions (11).

This study aims to determine the main dietary patterns related to multi-dimensional cognitive functions, such as global cognitive status, six cognitive domain functions, prevalence of MCI, and four main MCI subtypes in Chinese residents aged 55 years and older, using the data from the Community-based Cohort Study on Nervous System Diseases (CCSNSD).

## Materials and Methods

### Study Population

Community-based Cohort Study on Nervous System Diseases, a longitudinal study established by the National Institute for Nutrition and Health, Chinese Center for Disease Control and Prevention in 2018–2019, focuses on potential factors associated with risks of epilepsy, AD and Parkinson's disease (PD). We used a multistage stratified random sampling approach to draw participants without such diseases in Hebei, Zhejiang, Shaanxi, and Hunan provinces. Detailed survey design has been reported elsewhere (12). The Institutional Review Board of the National Institute for Nutrition and Health approved the study (No. 2017020, November 6, 2017). All participants provided their written informed consent.

This study recruited 6,143 eligible subjects aged 55 years and older without clinical diagnosis of AD or comorbidities. In the analysis, we used a subsample of 4,309 subjects (1,956 men and 2,353 women) aged between 55 and 86 years, excluding 1,769 subjects with incomplete data on sociodemographic characteristic, disease history, cognitive examination, food frequency questionnaire (FFQ), psychological evaluation, or basic abilities of daily living, and further excluding 65 subjects unable to perform basic activities of daily living, such as eating, dressing, bathing, toileting, grooming, moving to bed or chair, walking through the room, or incontinence.

### Identification of Dietary Patterns

We assessed dietary intake using a validated semi-quantitative FFQ covering 65 food items. Participants reported frequency categories (daily, weekly, monthly, annually, or never) and the average amount for each food item consumed in the last 12 months. We calculated their intake of each item based on the average intake frequency and quantity. If the subject reported the “never” intake frequency, we set his/her intake to 0 g/day or week. According to the participant's habitual intake and the similarity of types, we categorized 49 major food items into 13 subgroups, such as rice, wheat, tubers, legumes, fresh vegetables, fresh fruit, pork, beef and mutton, poultry, fish, eggs, dairy, and nuts. Supplementary Table S1 provides detailed information on the foods in each subgroup.

We used factor analysis with varimax rotation to analyze the 13 food subgroups and determined four factors, “meat-preferred” pattern, “plant-preferred” pattern, “eggs- and dairy-preferred” pattern, and “grain-preferred” pattern, based on eigenvalues >1.0, a scree plot, and the interpretability. Food items with an absolute factor load value >0.4 are the main contributors to the dietary pattern, representing the characteristics of each pattern. We calculated the factor scores of each participant for each pattern by summing the intake of food groups weighted by their factor loadings and grouped them into quartiles for further analysis. A higher quartile indicates more consistency with the pattern being calculated.

### Assessment of Cognitive Function

We used the Montreal Cognitive Assessment (MoCA) to assess the cognitive function of participants. The sum of the scale items produces a total MoCA score ranging from 0 to 30, which is positively associated with the global cognitive function (13). The MCI criteria is based on the Chinese MoCA norms (14): total MoCA score ≤ 13 for illiterate individuals, ≤ 19 for individuals with 1–6 years of education, and ≤ 24 for individuals with 7 years of education or more.

We evaluated the cognitive domain functions of memory, execution, visuospatial, language, attention, and orientation by the memory index score (MIS), executive index score (EIS), visuospatial index score (VIS), language index score (LIS), attention index score (AIS), and orientation index score (OIS), respectively. We calculated these index scores based on the MoCA cognitive domain index score (13). We defined each impaired cognitive domain as participants who scored <1.5 SDs below the age- and education-adjusted mean value in that cognitive domain (15). We defined MCI subtypes as participants having MCI and characterized by different cognitive domain deficits (16, 17): amnestic MCI single domain (aMCI-SD): only memory impairment; non-amnestic MCI single domain (naMCI-SD): a deficit in one cognitive domain other than memory; amnestic MCI multiple domains (aMCI-MD): memory impairment plus one other impaired domain; and non-amnestic MCI multiple domains (naMCI-MD): deficits in at least 2 domains other than memory.

### Assessment of Covariates

Interviewers with a degree in medicine or public health who have received two rounds of training by the national or provincial experts and passed the qualification examination collected all survey data, such as gender (male or female), age (years), education level (below primary school, primary school, or secondary school or above), residential area (rural or urban), current employment status (yes or no), and monthly household income per capita [ <1,000, 1,000–3,999, or ≥4,000 (Chinese yuan RMB)], smoking (never or ever/current), drinking (never or ever/current), physical activity (occupations, household chores, leisure time, and transportation activities), sleep duration (hours), and medication usage (yes or no), and other. We converted total physical activity hours into metabolic equivalent of tasks (METs) hours per week based on the Compendium of Physical Activities recommended by the American College of Sports Medicine Association (18), and divided them into tertiles (low, medium, and high). We categorized sleep hours based on the cutoff points recommended by the National Sleep Foundation, 7–9 h for participants aged between 55 and 64 years, and 7–8 h for participants 65 years and older (19). We calculated the total energy intake based on the China Food Composition Table.

We defined history of diet-related chronic diseases as participant having hypertension, diabetes, stroke, or myocardial infarction diagnosed or treated by physicians. We defined obesity as body mass index (BMI) ≥ 28 kg/m^2^, and central obesity as a waist circumference ≥90 cm for men and ≥85 cm for women. Detailed information on physical measurements has been reported elsewhere (12).

### Statistical Analysis

For continuous variables, we used mean ± SD to describe the distribution and Wilcoxon signed rank test or Kruskal–Wallis *H*-test to examine the differences among groups as appropriate. For categorical variables, we used quantity (percentage) and the chi-square test. We used a series of quantile regression models to estimate the associations of each dietary pattern with the global cognitive score and cognitive domain subtypes. We built a set of modes, such as model without any adjustment (Model 1), model adjusted for age, gender, residential area, education level, current employment status, and household income level (Model 2), model further adjusted for physical activity, smoking, drinking, sleep duration, and total food energy (Model 3), and model further adjusted for diet-related chronic disease history, obesity, and central obesity (Model 4). We used multivariable logistic regression models to calculate the adjusted odds ratio (*AOR*) and 95% *CI* to estimate the relationship between each dietary pattern and the prevalence of MCI and its subtypes, controlling for covariates including age, gender, residential area, education level, current employment status, household income level, physical activity, smoking, drinking, sleep duration, total energy intake, diet-related chronic disease history, obesity, and central obesity. In addition, we tested the linear trends for the prevalence of MCI and its subtypes by assigning the median value to the quartiles of each dietary pattern score as a continuous variable. We used SAS version 9.4 (SAS Institute, Inc., Cary, NC, USA) for all statistical analysis, and considered the statistical significance as a two-sided *p* < 0.05.

## Results

### Characteristics of Subjects

As shown in Table 1, the average age of the participants was 68.4 years. Among the 4,309 participants, 45.4% were men, 50.6% lived in rural areas, 85.8% achieved a primary education level or above, and 76.4% had a per capita monthly income more than 1,000 yuan. The proportion of participants with a history of diet-related chronic diseases, obesity, or central obesity were 36.3, 13.2, and 46.5%, respectively. Compared with participants with the normal cognitive function, MCI participants were older and poorer, and had lower energy intake (*p* < 0.05).

**Table 1:** Subject characteristics by cognitive status among Chinese adults aged 55 years and above in the Community-based Cohort Study on Nervous System Diseases (CCSNSD) 2018–2019.

**Characteristics**	***N* (%)**	**MCI**		***P-*value**
		**Yes**	**No**	
Total	4,309 (100.0)	42.6	57.4	
Age group (years)^a^				<0.001
55–64	1,586 (36.8)	38.1	61.9	
65–74	1,864 (43.3)	42.0	58.0	
≥75	859 (19.9)	51.9	48.1	
Gender^a^				0.476
Male	1956 (45.4)	42.0	58.0	
Female	2353 (54.6)	43.1	56.9	
Residential area^a^				<0.001
Urban	2,130 (49.4)	38.5	61.5	
Rural	2,179 (50.6)	46.5	53.5	
Education level^a^				<0.001
Illiteracy	613 (14.2)	39.0	61.0	
≤ Primary school	1,784 (41.4)	38.6	61.4	
≥Secondary school	1,912 (44.4)	47.4	52.6	
Current employment^a^				0.011
Yes	772 (17.9)	38.5	61.5	
No	3,537 (82.1)	43.5	56.5	
Monthly household income per capital (RMB)^a^				<0.001
<1,000	1,017 (23.6)	54.2	45.8	
1,000–3,999	2,653 (61.6)	42.2	57.8	
≥4,000	639 (14.8)	25.5	74.5	
Physical activity level^a^				<0.001
Low	1,431 (33.2)	38.1	61.9	
Medium	1,442 (33.5)	43.7	56.3	
High	1,436 (33.3)	45.9	54.1	
Smoking^a^				0.006
Ever/current	1,036 (24.0)	46.2	53.8	
Never	3,273 (76.0)	41.4	58.6	
Drinking^a^				0.214
Ever/current	743 (17.2)	40.5	59.5	
Never	3,566 (82.8)	43.0	57.0	
Meeting sleep duration recommendation^a^				0.187
Yes	1,505 (34.9)	43.9	56.1	
No	2,804 (65.1)	41.8	58.2	
Medical history^a^				0.004
Yes	1,562 (36.3)	45.5	54.5	
No	2,747 (63.7)	40.9	59.1	
Obesity^a^				0.165
Yes	568 (13.2)	45.2	54.8	
No	3,741 (86.8)	42.2	57.8	
Central obesity^a^				0.969
Yes	2,005 (46.5)	42.6	57.4	
No	2,304 (53.5)	42.5	57.5	
Energy^b^	1,522.0 ± 620.7	1,484.3 ± 592.9	1,549.8 ± 639.3	0.003

a *Values are expressed as N (%) or % and examined using chi-square test*.

b *Values are expressed as mean ± SD (kcal) and examined using Wilcoxon signed rank test*.

### Dietary Patterns

We identified four dietary patterns using factor analysis. Table 2 presents the factor load in each food group. These four patterns explained 50.1% of the variance in dietary intake (i.e., 23.0% by factor 1, 10.9% by factor 2, 8.9% by factor 3, and 7.3% by factor 4). The first factor “meat-preferred” pattern, was characterized by a large intake of pork, fish, poultry, and beef or lamb. The second pattern, “plant-preferred” pattern, included a large intake of tubers, legumes, fresh vegetables, fresh fruit, and nuts. The third pattern, “eggs- and dairy-preferred” pattern, referred to a large intake of eggs and dairy products. The fourth pattern, “grain-preferred” pattern, was characterized by a large intake of rice- and wheat-based foods. The intake of each food group was significantly different among the quartiles of each dietary pattern (*p* < 0.05, Supplementary Table S2).

**Table 2:** Factor load in food groups of dietary patterns^a^.

**Food groups**	**Meat-preferred pattern**	**Plant-preferred pattern**	**Eggs- and dairy-preferred pattern**	**Grain-preferred pattern**
	**(factor 1)**	**(factor 2)**	**(factor 3)**	**(factor 4)**
Pork	**0.773**	−0.029	0.040	0.020
Fish	**0.701**	0.153	−0.119	−0.020
Poultry	**0.448**	0.330	0.245	0.002
Beef or mutton	**0.445**	0.212	0.342	−0.041
Tubers	−0.034	**0.716**	−0.029	0.148
Legumes	0.090	**0.605**	0.317	−0.101
Fresh vegetables	0.520	**0.573**	0.116	0.071
Fresh fruit	0.183	**0.539**	0.353	0.055
Nuts	0.112	**0.441**	−0.052	−0.078
Eggs	0.218	−0.053	**0.719**	0.186
Dairy	−0.160	0.191	**0.615**	−0.174
Wheat	−0.294	0.034	0.243	**0.742**
Rice	0.217	0.001	−0.192	**0.703**

a *The absolute value of factor load was >0.4 is shown in bold. For food group load with more than one dietary pattern, only the highest absolute value of the load is shown in bold*.

Supplementary Table S3 presents sociodemographic status, lifestyle variables, and health-related factors for each quartile of dietary pattern scores. The distributions of residential area, education level, employment status, household income level, drinking, and energy intake were significantly different among the quartiles of the four dietary patterns (*p* < 0.05). Physical activity and smoking status were significantly different among the quartiles of the “meat-preferred” pattern, the “plant-preferred” pattern, and the “grain-preferred” pattern (*p* < 0.05). The history of diet-related chronic diseases, obesity, and central obesity were significantly different among the quartiles of the “meat-preferred” pattern and the “eggs- and dairy-preferred” pattern (*p* < 0.05).

### Dietary Patterns and Global Cognition and Cognitive Domains

Table 3 presents the global cognitive score and cognitive domain subscores of the quartiles of each dietary pattern. Participants in the highest quartile of the “meat-preferred” pattern had higher global cognitive function scores and higher scores in most cognitive domains (*p* < 0.05) compared with those in other quartiles. The global cognitive function scores and cognitive domains were significantly increased as the “plant-preferred” pattern score increased from the first quartile to the fourth quartile (*p* < 0.05), while they were decreased as the “grain-preferred” pattern score from the lowest to the highest quartile (*p* < 0.05). The lowest and the highest scores of global cognitive function, execution, visuospatial, language, and attention were distributed in the third quartile and the fourth quartile of the “eggs- and dairy-preferred” pattern (*p* < 0.05).

**Table 3:** Differences in global cognitive score and cognitive domain subscores by quartiles of each dietary pattern score^a^.

**Dietary patterns**	**Global cognitive function**	**Cognitive domain scores**					
		**MIS**	**EIS**	**VIS**	**LIS**	**AIS**	**OIS**
Meat-preferred pattern							
Q1	20.22 ± 6.06	10.48 ± 4.46	8.00 ± 3.26	5.06 ± 1.73	4.38 ± 1.40	12.52 ± 4.18	5.31 ± 1.17
Q2	20.82 ± 6.27	10.42 ± 4.43	8.47 ± 3.31	5.20 ± 1.70	4.38 ± 1.47	12.54 ± 4.11	5.46 ± 1.04
Q3	22.28 ± 6.36	11.38 ± 4.10	9.09 ± 3.37	5.46 ± 1.76	4.58 ± 1.43	13.58 ± 3.99	5.57 ± 0.92
Q4	22.80 ± 6.00	12.00 ± 3.67	9.22 ± 3.35	5.33 ± 1.89	4.65 ± 1.36	14.00 ± 3.60	5.63 ± 0.78
*p-*value	<0.001	<0.001	<0.001	<0.001	<0.001	<0.001	<0.001
Plant-preferred pattern							
Q1	20.26 ± 6.67	10.51 ± 4.41	8.09 ± 3.60	4.92 ± 1.83	4.25 ± 1.49	12.34 ± 4.25	5.32 ± 1.11
Q2	20.86 ± 6.27	10.70 ± 4.50	8.38 ± 3.31	5.09 ± 1.84	4.41 ± 1.42	12.89 ± 3.94	5.41 ± 1.08
Q3	21.83 ± 6.00	11.35 ± 3.96	8.76 ± 3.25	5.41 ± 1.73	4.56 ± 1.39	13.26 ± 4.01	5.56 ± 0.92
Q4	23.17 ± 5.66	11.72 ± 3.91	9.55 ± 3.06	5.64 ± 1.62	4.78 ± 1.32	14.15 ± 3.69	5.68 ± 0.81
*p-*value	<0.001	<0.001	<0.001	<0.001	<0.001	<0.001	<0.001
Eggs- and dairy-preferred pattern							
Q1	21.83 ± 6.60	11.25 ± 4.25	8.93 ± 3.44	5.09 ± 1.98	4.44 ± 1.51	13.46 ± 3.96	5.54 ± 0.95
Q2	21.26 ± 5.98	11.04 ± 4.14	8.46 ± 3.27	5.28 ± 1.71	4.47 ± 1.37	12.97 ± 3.92	5.50 ± 0.95
Q3	20.67 ± 6.23	10.74 ± 4.24	8.16 ± 3.35	5.12 ± 1.76	4.38 ± 1.44	12.65 ± 4.18	5.43 ± 1.07
Q4	22.36 ± 6.09	11.25 ± 4.27	9.23 ± 3.26	5.57 ± 1.61	4.70 ± 1.34	13.56 ± 3.98	5.51 ± 1.00
*p*-value	<0.001	<0.001	<0.001	<0.001	<0.001	<0.001	0.160
Grain-preferred pattern							
Q1	22.96 ± 5.89	11.63 ± 3.79	9.44 ± 3.17	5.69 ± 1.59	4.79 ± 1.33	13.71 ± 3.87	5.66 ± 0.82
Q2	21.77 ± 6.31	11.26 ± 4.15	8.78 ± 3.38	5.21 ± 1.84	4.46 ± 1.44	13.36 ± 3.91	5.58 ± 0.88
Q3	21.35 ± 6.23	10.92 ± 4.32	8.61 ± 3.34	5.16 ± 1.86	4.40 ± 1.46	13.16 ± 4.04	5.55 ± 0.95
Q4	20.05 ± 6.26	10.45 ± 4.53	7.94 ± 3.36	5.00 ± 1.73	4.35 ± 1.40	12.41 ± 4.18	5.19 ± 1.22
*p*-value	<0.001	<0.001	<0.001	<0.001	<0.001	<0.001	<0.001

a *MIS, memory index score; EIS, executive index score; VIS, visuospatial index score; LIS, language index score; AIS, attention index score; OIS, orientation index score. Q1–Q4 are quartiles of each dietary pattern score. Global cognitive function score and cognitive domain subscores are expressed as mean ± SD, evaluated by Montreal Cognitive Assessment (MoCA, Beijing Version) and examined using Kruskal–Wallis H-test*.

Table 4 presents the association of the four dietary patterns with the global cognitive score and cognitive domain subscores. In the final multivariate models, the average scores of the global cognition, memory, execution, and attention were increased significantly as the quartiles of the “meat-preferred” pattern score increased (*p* < 0.05); similarly, participants with high intake of the “plant-preferred” pattern may have higher scores of 0.45, 0.25, 0.18, 0.13, 0.07, and 0.30 in the global cognition, memory, execution, visuospatial, language, and attention function (*p* < 0.05), respectively. In contrast, the average scores of global cognition, execution, visuospatial, and language function were decreased significantly as the quartiles of the “grain-preferred” pattern score increased (*p* < 0.05). After adjusting for all potential factors, only a positively significant relationship was observed between the visuospatial function score and the “eggs- and dairy-preferred” pattern (*p* < 0.05). No significant association was observed between the orientation function score and any of the four dietary patterns (*p* > 0.05).

**Table 4:** Quantile regression analysis of associations of four dietary pattern scores with global cognitive score and cognitive domain subscores^a^.

**Dietary patterns**	**Global cognitive function**		**Cognitive domain scores**											
	**β**	***p-*value**	**MIS**		**EIS**		**VIS**		**LIS**		**AIS**		**OIS**	
			**β**	***p-*value**	**β**	***p-*value**	**β**	***p-*value**	**β**	***p-*value**	**β**	***p-*value**	**β**	***p-*value**
Meat-preferred pattern														
Model 1	1.00	<0.001	0.50	<0.001	0.50	<0.001	0.00	1.000	0.00	1.000	0.67	<0.001	0.00	1.000
Model 2	0.43	<0.001	0.25	<0.001	0.15	0.001	0.00	1.000	0.00	0.814	0.25	<0.001	0.00	1.000
Model 3	0.42	<0.001	0.23	<0.001	0.12	0.034	−0.01	0.576	0.00	0.999	0.26	<0.001	0.00	0.986
Model 4	0.42	<0.001	0.20	<0.001	0.11	0.021	−0.01	0.669	0.01	0.653	0.22	<0.001	0.00	0.994
Plant-preferred pattern														
Model 1	1.00	<0.001	0.33	<0.001	0.50	<0.001	0.00	1.000	0.00	1.000	0.50	<0.001	0.00	1.000
Model 2	0.40	<0.001	0.17	0.012	0.17	<0.001	0.14	<0.001	0.00	0.957	0.32	<0.001	0.00	1.000
Model 3	0.47	<0.001	0.22	<0.001	0.18	0.001	0.15	<0.001	0.06	0.035	0.30	<0.001	0.00	1.000
Model 4	0.45	<0.001	0.25	<0.001	0.18	<0.001	0.13	<0.001	0.07	0.008	0.30	<0.001	0.00	1.000
Eggs- and dairy-preferred pattern														
Model 1	0.00	1.000	0.00	1.000	0.00	1.000	0.00	1.000	0.00	1.000	0.00	1.000	0.00	1.000
Model 2	0.00	1.000	0.00	0.976	−0.04	0.389	0.08	<0.001	0.00	0.817	0.00	0.968	0.00	1.000
Model 3	0.00	0.983	0.00	0.981	−0.04	0.403	0.07	0.003	0.03	0.228	0.00	0.991	0.00	1.000
Model 4	0.05	0.568	0.04	0.412	−0.04	0.395	0.08	0.002	0.04	0.056	−0.02	0.758	0.00	1.000
Grain-preferred pattern														
Model 1	−1.00	<0.001	−0.33	<0.001	−0.50	<0.001	0.00	1.000	0.00	1.000	−0.50	<0.001	0.00	1.000
Model 2	−0.50	<0.001	0.00	0.977	−0.23	<0.001	−0.11	<0.001	0.00	0.970	0.00	0.997	0.00	1.000
Model 3	−0.35	<0.001	0.00	0.998	−0.22	<0.001	−0.10	<0.001	−0.03	0.222	−0.12	0.048	0.00	0.986
Model 4	−0.36	<0.001	0.00	0.911	−0.19	0.001	−0.09	<0.001	−0.05	0.039	−0.11	0.071	0.00	0.894

a *MIS, memory index score; EIS, executive index score; VIS, visuospatial index score; LIS, language index score; AIS, attention index score; OIS, orientation index score. Global cognitive function score and cognitive domain subscores are evaluated by MoCA (Beijing Version). Model 1 was unadjusted; Model 2 adjusted for age, gender, residential area, education level, current employment status, and household income level; Model 3 further adjusted for physical activity, smoking, drinking, sleep duration, and total food energy; and Model 4 further adjusted for diet-related chronic disease history, obesity, and central obesity*.

### Dietary Patterns and MCI and Its Subtypes

**Figure 1:**
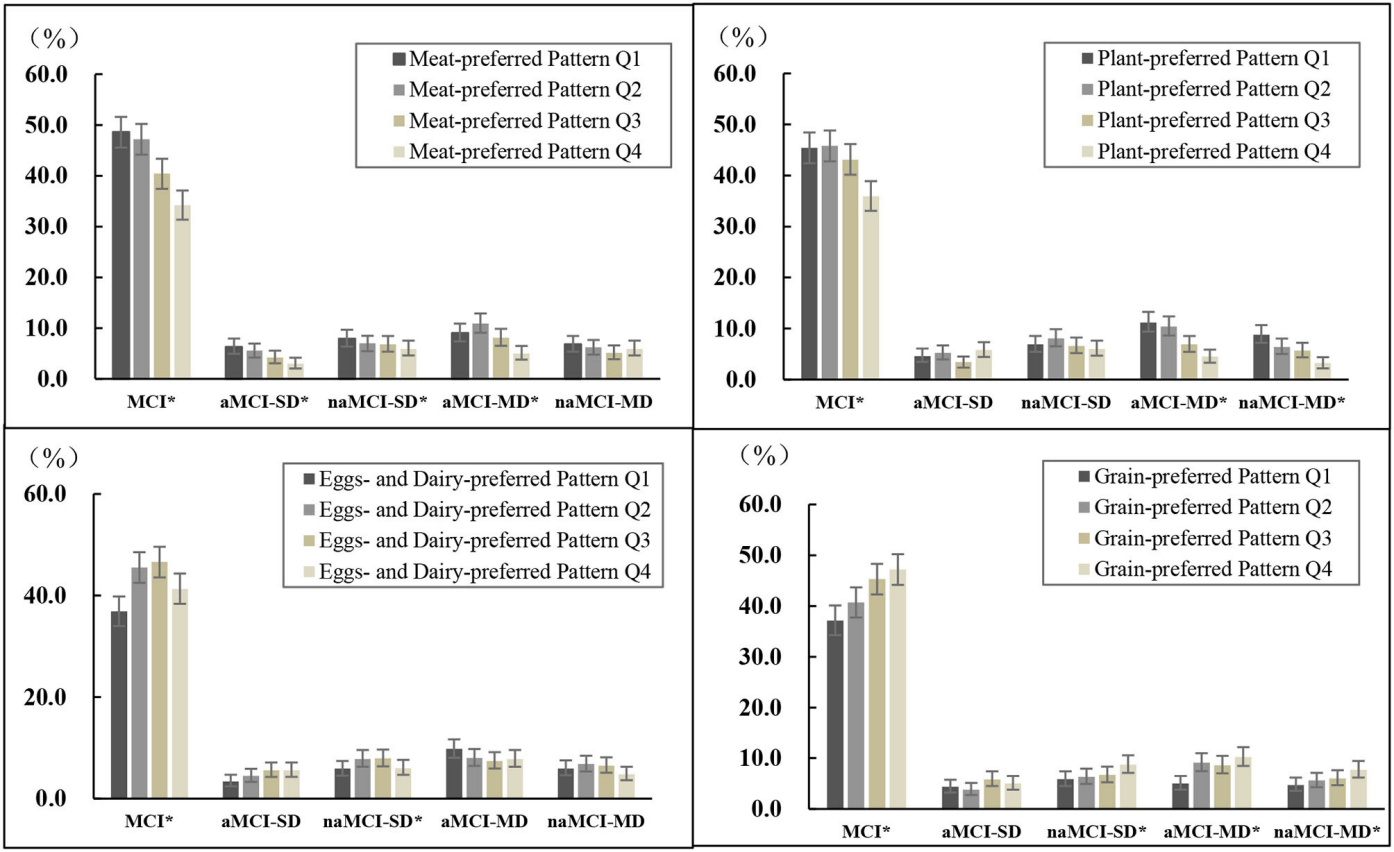
Prevalence of MCI and its subtypes by quartiles of each dietary pattern score^a^. ^a^Q1–Q4 are quartiles of each dietary pattern score. MCI, mild cognitive impairment; aMCI-SD, amnestic MCI single domain; naMCI-SD, non-amnestic MCI single domain; aMCI-MD, amnestic MCI multiple domains; naMCI-MD, non-amnestic MCI multiple domains. *p < 0.05 within dietary pattern score category, examined by chi-square test.

As shown in Figure 1, the prevalence distributions of MCI and some of its subtypes differed among the quartiles of each dietary pattern. Participants in the highest quartile of the “meat-preferred” pattern had lower prevalence of MCI, aMCI-SD, naMCI-SD, and aMCI-MD (*p* < 0.05); and similarly, those in the highest quartile of the “plant-preferred” pattern had lower prevalence of MCI, aMCI-MD, and naMCI-MD (*p* < 0.05), compared with those in other quartiles of the corresponding pattern. In contrast, participants in the bottom quartile of the “eggs- and dairy-preferred” pattern had lower prevalence of MCI, aMCI-SD, and naMCI-SD (*p* < 0.05), and those in the bottom quartile of the “grain-preferred” pattern had lower prevalence of MCI, naMCI-SD, aMCI-MD, and naMCI-MD (*p* < 0.05).

**Figure 2:**
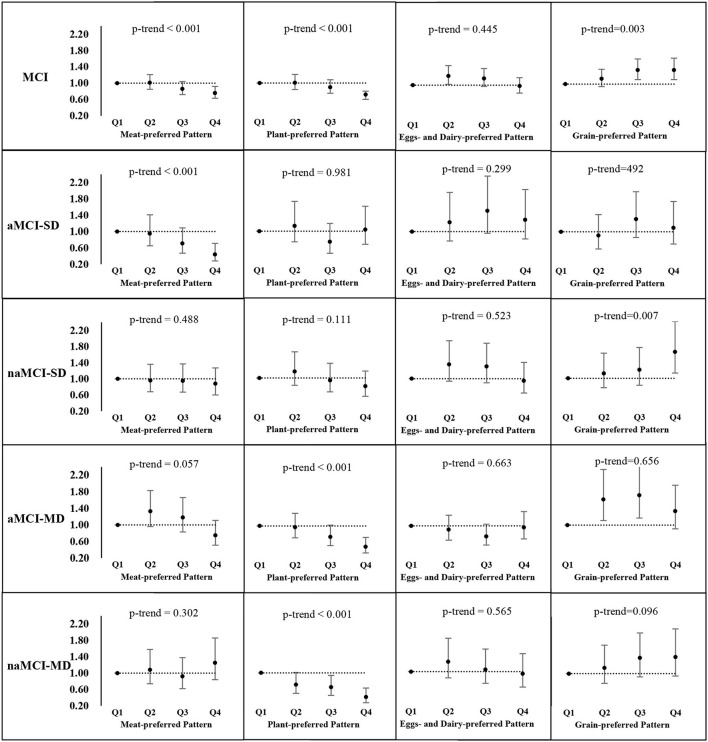
Adjusted odds ratios (*AOR*) and the corresponding 95% *CI*s for MCI and its subtypes across quartiles of each dietary pattern score^a^. ^a^MCI, mild cognitive impairment; aMCI-SD, amnestic MCI single domain; naMCI-SD, non-amnestic MCI single domain; aMCI-MD, amnestic MCI multiple domains; naMCI-MD, non-amnestic MCI multiple domains. Adjusted for age, gender, residential area, education level, current employment status, household income level, physical activity, smoking, drinking, sleep duration, total food energy, diet-related chronic disease history, obesity, and central obesity.

Figure 2 presents *AOR* and the 95% *CI* of MCI and its subtypes in the quartiles of each dietary pattern score. After controlling for multi-covariates, participants in the highest quartile of the “meat-preferred” pattern had 24 and 56% lower odds of MCI (*AOR* = 0.76, 95% *CI* 0.63–0.92) and aMCI-SD (*AOR* = 0.44, 95% *CI* 0.28–0.71), respectively, compared with those in the lowest quartile; similarly, those in the highest quartile of the “plant-preferred” pattern were less likely to have MCI, aMCI-MD, and naMCI-MD. The *AOR* was 0.72 (95% *CI* 0.60–0.80), 0.49 (95% *CI* 0.34–0.72), and 0.41 (95% *CI* 0.27–0.63), respectively. In addition, the increase in each quartile level of these pattern scores significantly reduced the odds of MCI and the corresponding subtypes mentioned above (*p-trend* < 0.05). On the contrary, participants in the highest quartile of the “grain-preferred” pattern score tended to have 34% (*AOR* = 1.34, 95% *CI* 1.11–1.63, *p-trend* = 0.003) and 65% (*AOR* = 1.65, 95% *CI* 1.13–2.40, *p-trend* = 0.007) higher odds of MCI and naMCI-SD compared with those in the lowest quartile, and those in the second quartile of the “eggs- and dairy-preferred” pattern score had an AOR of 1.22 (95% *CI* 1.02–1.47) for the prevalence of MCI.

## Discussion

In this cross-sectional study, we identified four different dietary patterns: “meat-preferred” pattern (pork, fish, poultry, and beef or mutton), “plant-preferred” pattern (tubers, legumes, fresh vegetables, fresh fruit, and nuts), “eggs- and dairy-preferred” pattern (eggs and dairy products), and “grain-preferred” pattern (rice- and wheat-based foods). We found that adherence to the “meat-preferred” pattern and the “plant-preferred” pattern increased the scores of the global cognitive function and several cognitive domain functions and decreased the odds of MCI and some subtypes after adjustments for all potential confounding variables. Conversely, better adherence to the “grain-preferred” pattern reduced cognitive function scores and increased the odds of MCI and naMCI-SD in adjusted models. In addition, we observed that the “eggs- and dairy-preferred” pattern was associated with higher cognitive domain scores of visuospatial function. Participants in the second quartile of this pattern score had a 22% (95% *CI*: 1.02–1.47) higher odds of MCI compared with those in the bottom quartile.

In this study, the “meat-preferred” pattern is conducive in improving the cognitive function and reducing the odds of MCI, aMCI-MD, and naMCI-MD, supported by its food intake profile that is characterized by high intake of pork, fish, poultry, beef, and lamb. Previous studies have showed that these foods play a potentially beneficial role in cognition (12, 20). Specifically, we found that participants in the third or top quartile intake of pork, beef or mutton, poultry, and fish had about 20–30% lower *OR*s of MCI and its some subtypes, respectively, compared with those in the bottom intake of the corresponding food group (12). Moreover, the results of a large longitudinal study of 5,934 French people 65 years and older with an average follow-up of 9.8 years showed that the low-meat intake is associated with an increased risk of cognitive impairment compared with regular intake frequency (7). In fact, some researchers thought that the high-meat intake with high saturated fat content increases inflammation and circulating cytokines, and have a negative impact on cognition (21), but this may be offset by the protein source itself which is beneficial to cognitive function by promoting the production and release of catecholamines, when maintaining a reasonable balance of this pattern. In addition, increasing the intake of fish rich in polyunsaturated fatty acids could significantly combat the pathologic cognitive impairment of the elderly shown by mechanism studies and epidemiologic analyses (22, 23), which supports that this pattern is related to superior cognitive function.

Our findings show that the “plant-preferred” pattern is positively associated with a variety of cognitive functions ranging from the global mental status to specific domains. Food groups that are good for to brain are fruit, vegetables, legumes, and nuts, which are rich in fiber, beta carotene, vitamins K and C, folate, and especially magnesium (24, 25). These antioxidants, which are considered to have anti-inflammatory properties, have shown to be significantly associated with the prevention of cognitive impairment (26), because oxidative stress and inflammation are the triggers of the cognitive decline process (27). Some epidemiological studies are consistent with the above findings, despite the dietary pattern analysis is different (28, 29). For example, a food pattern related to high intake of vegetables, fruit, legumes, and nuts, derived through a reduced rank regression analysis of 2,311 Chinese elderly adults aged 60–88 years, is associated with better memory and language function and a lower likelihood of MCI (29). Although the main criticism of plant-based diets is the risk of vitamin B_12_ deficiency (30, 31), which is correlated to cognitive dysfunction (e.g., fatigue, memory, and movement problems) (32). However, moderate intake of poultry, beef or lamb can provide efficient vitamin B_12_ intake. This is another important feature of the “plant-preferred” pattern in this study, providing a plausible explanation to our findings.

Eggs, milk, and dairy products are nutritious foods that contain a variety nutrients associated with improving cognitive function, such as folate, vitamin B_12_ (33), choline (34), and protein (35). In 2019, a systematic review of six studies summarized the effects of milk and dairy products intake on cognitive function, indicating that dairy products may help prevent cognitive decline (36). However, a study of 3,835 American adults 65 years conducted in the same year did not find a similar relationship between egg intake and cognitive health (37). Our study found that eggs and dairy foods intake is positively correlated with higher scores of the “eggs- and dairy-preferred” pattern, which may improve visuospatial ability. In addition, we observed that the intake of eggs in the third quartile of this pattern score reached the recommended amount (40–50 g/day) of the Chinese Dietary Guidelines, but even in the top quartile the dairy intake did not reach its recommended level (300 g/day). In this regard, the difference in the intake of eggs and dairy foods intakes in the first two quartiles of the pattern is not significant. Therefore, the observation that subjects in the second quartile tended to have higher odds of MCI than subjects in the bottom quartile may be caused by data variances.

In addition, in our study, adults who adhere to the “grain-preferred” pattern consume more rice- and wheat-based foods, and their cognitive functions are often incomplete, which is consisting with the findings in the literature. There is evidence that high glycemic index foods may cause cognitive impairment through adverse effects on neuronal integrity and glucose metabolism in the nervous system (38, 39). It is worth noting that there is evidence that moderate intake of rice is negatively associated with cognitive functions (12, 40). Given that wheat intake increased as the quartiles of this pattern score increased, but rice intake did not increase, although both intakes were dominant. Therefore, we speculate that the detrimental effects of this pattern on cognition may be mainly due to the large intake of wheat-based foods. Of course, large-scale prospective cohort studies are needed to provide more clues to the underlying mechanism.

This study has several limitations. First, the cross-sectional nature of our study cannot provide evidence of any causal conclusions between dietary patterns and multidimensional cognition. It must consider the possibility of reverse causality that changes in dietary patterns lead to cognitive decline that cannot be captured. Second, the intake of food groups comes from a valid, semi-quantitative FFQ covering 1 year. Ranking subjects based on the food intake and presenting the profiles information of dietary nutrients intake in patterns is not completely accurate and may also lead to recall bias. However, the census concluded that recall errors due to cognitive impairment are believed to bias the results toward the null hypothesis. Third, the factor analysis method is highly data-driven, and thus dominant food groups are still interacted with other second or minor food groups. Fourth, although we carefully adjusted some covariates during the data analysis process, we may not consider residual confounding factors (e.g., hyperlipidemia, social engagement, living conditions, etc.). Finally, because this study covers adults in four provinces of China only, any generalization of the results we obtained to other regions should be carried out with caution. However, the CCSNSD is specifically designed to investigate the risk factors of neurological diseases in China. The population-based design reduces the selection bias, and a comprehensive evaluation of participant's cognitive function by trained evaluators with a degree in medicine or public health increases the internal validity of the findings.

This study showed the associations of four distinct dietary patterns with cognitive functions among Chinese adults, and thus findings can contribute to the establishment of dietary guidelines targeting older Chinese adults to reduce the cognitive impairment. It is of great significance to provide upgraded scientific evidence and strategies for early nutrition intervention and have important theoretical and social significance for promoting healthy aging.

## Conclusion

To conclude, the study is the first to examine the relationship between dietary patterns and multidimensional cognitive function, global cognitive status, six cognitive domain functions, and cognitive-related outcomes of MCI and its four subtypes in Chinese adults. The study showed that the “plant-preferred” pattern and the “meat-preferred” pattern are associated with higher cognitive function scores and lower odds of MCI and some of its subtypes, which further provide evidence that higher intake of diet rich in vegetables, fruit, legumes, nuts, and meat may help prevent cognitive decline, and people who prefer grain foods, especially wheat-based foods, are negatively associated with cognitive health. However, the exact role of the “eggs- and dairy-preferred” pattern in cognition remains to be determined. Future prospective cohort studies are needed to examine the effects of these patterns on brain health to prove the causal relationship between dietary patterns and cognitive function.

## Data Availability Statement

The raw data supporting the conclusions of this article will be made available by the authors, without undue reservation.

## Ethics Statement

The studies involving human participants were reviewed and approved by the Institutional Review Board of the National Institute for Nutrition and Health (No. 2017020, November 6, 2017). The patients/participants provided their written informed consent to participate in this study.

## Author Contributions

QH, XJ, JZ, and ZW contributed to the conceptualization of the manuscript. QH and JZ performed the methodology. QH is responsible for the formal analysis and writing—original draft preparation. FH performed data curation. XJ, JZ, and ZW contributed to writing—review. ZW and BZ contributed to project administration. HW is responsible for supervision. FH, LW, HJ, MG, YH, WS, YM, and XZ contributed to the investigation. All authors read and approved the final manuscript.

## Funding

This study was supported by grants from the Ministry of Finance of China, the National Key R&D Program of China, the Precision Medicine Project–Cohort Study on Nervous System Diseases (2017YFC0907700), and the Community-based Cohort Study on Nervous System Diseases (2017YFC0907701).

## Conflict of Interest

The authors declare that the research was conducted in the absence of any commercial or financial relationships that could be construed as a potential conflict of interest.

## Publisher's Note

All claims expressed in this article are solely those of the authors and do not necessarily represent those of their affiliated organizations, or those of the publisher, the editors and the reviewers. Any product that may be evaluated in this article, or claim that may be made by its manufacturer, is not guaranteed or endorsed by the publisher.

## Abbreviations

AIS: Attention index score
EIS: Executive index score
LIS: Language index score
MCI: Mild cognitive impairment
MIS: Memory index score
OIS: Orientation index score
VIS: Visuospatial index score
aMCI-SD: Amnestic MCI single domain
aMCI-MD: Amnestic MCI multiple domains
naMCI-SD: Non-amnestic MCI single domain
naMCI-MD: Non-amnestic MCI multiple domains.
