# Supplementary material for: Dietary Patterns Are Associated With Multi-Dimensional Cognitive Functions Among Adults Aged 55 and Older in China
Source: Front Nutr. 2022 Feb 17;9:806871. doi: 10.3389/fnut.2022.806871 (PMC8891750; doi:10.3389/fnut.2022.806871)
Supplement: Supplementary file 1 [file Table_1.DOCX]

| Table S1 Food grouping used in the dietary pattern analysis | |
| --- | --- |
| Food group | Detailed food items |
| Rice | Cooked white rice, cooked rice with assorted mixtures, rice-noodle |
| Wheat | Non-fried noodles (white bread, steamed buns, noodles, dumplings) |
| Tubers | Sweet potato, potato, taro |
| Legumes | Soybeans, mung beans, red beans, soy milk, soy flour, tofu |
| Fresh vegetables | Fresh legume vegetables, tomatoes, peppers, melon vegetables, green leafy vegetables, cabbage and other leafy vegetables, cruciferous vegetables |
| Fresh fruit | Orange fruits, melon fruits, berry fruits, all other fresh fruits |
| Pork | Lean pork, fatty pork |
| Beef or mutton | Beef, lamb, mutton |
| Poultry | Chicken, duck, goose, pigeon, quail |
| Fish | Sea fish, freshwater fish |
| Eggs | Fresh egg, salted egg, preserved egg |
| Dairy | Liquid milk, milk powder, yogurt, cheese |
| Nuts | Peanuts, melon seeds, pumpkin seeds, watermelon seeds |

Table S2 Food intakes across the quartiles of each dietary pattern ^a^

| Food groups | Q1(n=1077) | | | Q2(n=1077) | Q3(n=1078) | Q4(n=1077) |
| --- | --- | --- | --- | --- | --- | --- |
| **Meat-preferred Pattern** | | |  |  |  |  |
| Rice (g/day) * | 28.6 (6.7, 71.4) | | | 100.0 (21.4, 200.0) | 150.0 (57.9, 250.0) | 200.0 (150.0, 300.0) |
| Wheat (g/day) * | 200.0 (100.0, 300.0) | | | 80.0 (28.6, 150.0) | 57.1 (14.3, 100.0) | 42.9 (14.3, 100.0) |
| Tubers (g/day) * | 28.6 (14.3, 50.0) | | | 14.3 (4.0, 34.3) | 16.2 (5.7, 37.1) | 20.6 (8.0, 49.5) |
| Legumes (g/day) * | 31.4 (12.9, 80.5) | | | 18.6 (6.7, 50.5) | 28.6 (11.9, 62.1) | 40.7 (21.1, 85.7) |
| Fresh vegetables (g/day) * | 96.7 (55.2, 185.7) | | | 98.3 (53.8, 171.4) | 197.0 (115.4, 300.0) | 310.7 (223.2, 445.2) |
| Fresh fruit (g/day) * | 33.5 (9.6, 82.9) | | | 25.0 (10.6, 57.1) | 42.9 (21.4, 92.8) | 60.3 (33.6, 108.3) |
| Pork (g/day) * | **7.1 (2.0, 14.3)** | | | **14.3 (4.7, 25.7)** | **28.6 (17.1, 50.0)** | **100.0 (50.0, 160.0)** |
| Beef or mutton (g/day) * | **0.0 (0.0, 1.4)** | | | **0.3 (0.0, 2.2)** | **1.6 (0.0, 6.7)** | **6.7 (1.2, 10.0)** |
| Poultry (g/week) * | **8.4 (0.0, 32.5)** | | | **18.7 (1.9, 50.0)** | **46.7 (16.3, 100.0)** | **93.0 (38.4, 200.0)** |
| Fish (g/week) * | **7.0 (0.0, 30.3)** | | | **14.0 (0.0, 46.7)** | **70.0 (23.3, 140.0)** | **250.0 (116.7, 400.0)** |
| Eggs (g/day) * | 25.7 (14.3, 50.0) | | | 21.4 (8.6, 50.0) | 27.2 (14.3, 51.7) | 30.4 (15.0, 60.0) |
| Dairy (g/day) * | 28.6 (0.0, 142.9) | | | 0.0 (0.0, 47.1) | 0.0 (0.0, 47.0) | 0.0 (0.0, 17.9) |
| Nuts(g/week) * | 0.0 (0.0, 14.0) | | | 0.0 (0.0, 14.0) | 5.8 (0.0, 50.0) | 16.3 (0.0, 70.0) |
| **Plant-preferred Pattern** | |  | |  |  |  |
| Rice (g/day) * | 150.0 (28.6, 300.0) | | | 100.0 (20.0, 225.0) | 100.0 (28.6, 180.0) | 100.0 (42.9, 200.0) |
| Wheat (g/day) * | 75.0 (17.1, 200.0) | | | 65.0 (25.7, 150.0) | 87.9 (28.6, 150.0) | 100.0 (42.9, 150.0) |
| Tubers (g/day) * | **5.3 (0.0, 12.6)** | | | **14.3 (7.1, 25.7)** | **28.6 (15.0, 43.7)** | **57.1 (28.6, 100.0)** |
| Legumes (g/day) * | **10.5 (2.5, 28.6)** | | | **20.0 (8.8, 37.0)** | **42.9 (20.8, 77.1)** | **89.0 (43.1, 151.4)** |
| Fresh vegetables (g/day) * | **88.7 (42.6, 179.0)** | | | **126.7 (71.0, 227.3)** | **183.6 (108.8, 287.2)** | **328.8 (187.8, 488.1)** |
| Fresh fruit (g/day) * | **19.3 (6.7, 41.7)** | | | **28.6 (12.8, 55.0)** | **50.7 (24.7, 89.0)** | **99.0 (50.0, 180.0)** |
| Pork (g/day) * | 22.4 (7.0, 64.3) | | | 17.1 (6.7, 42.9) | 21.4 (8.0, 50.0) | 28.6 (11.4, 57.1) |
| Beef or mutton (g/day) * | 0.2 (0.0, 2.9) | | | 0.7 (0.0, 3.3) | 1.0 (0.0, 6.7) | 3.3 (0.0, 10.0) |
| Poultry (g/week) * | 16.3 (0.0, 46.7) | | | 23.3 (5.8, 58.3) | 39.2 (3.8, 100.0) | 80.0 (23.3, 189.0) |
| Fish (g/week) * | 23.3 (0.0, 140.0) | | | 35.0 (3.8, 100.0) | 41.9 (3.8, 111.9) | 93.3 (23.3, 200.0) |
| Eggs (g/day) * | 37.0 (11.4, 60.0) | | | 21.4 (8.7, 49.5) | 22.8 (14.3, 50.0) | 34.3 (17.0, 60.0) |
| Dairy (g/day) * | 0.0 (0.0, 6.7) | | | 0.0 (0.0, 28.6) | 2.5 (0.0, 80.0) | 29.2 (0.0, 114.3) |
| Nuts(g/week) * | **0.0 (0.0, 11.5)** | | | **1.7 (0.0, 14.4)** | **5.8 (0.0, 56.0)** | **35.0 (0.0, 150.0)** |
| **Eggs and Dairy Pattern** | |  | |  |  |  |
| Rice (g/day) * | 220.0 (150.0, 300.0) | | | 100.0 (21.4, 200.0) | 57.1 (21.4, 150.0) | 68.6 (21.4, 150.0) |
| Wheat (g/day) * | 25.7 (6.7, 80.0) | | | 80.0 (28.6, 150.0) | 100.0 (50.0, 200.0) | 102.9 (60.0, 240.0) |
| Tubers (g/day) * | 13.0 (4.3, 35.7) | | | 19.8 (7.6, 38.6) | 25.7 (10.5, 45.2) | 21.4 (7.1, 48.6) |
| Legumes (g/day) * | 16.1 (4.4, 38.0) | | | 28.6 (13.3, 57.1) | 35.7 (14.3, 72.8) | 56.7 (20.0, 118.6) |
| Fresh vegetables (g/day) * | 171.4 (75.2, 301.4) | | | 132.9 (71.4, 262.3) | 154.3 (87.9, 262.5) | 209.0 (101.4, 354.6) |
| Fresh fruit (g/day) * | 25.4 (11.0, 56.0) | | | 31.7 (13.5, 62.7) | 42.8 (21.0, 84.6) | 85.7 (35.7, 164.3) |
| Pork (g/day) * | 25.7 (8.7, 57.7) | | | 21.4 (7.5, 49.5) | 20.0 (6.7, 50.0) | 28.6 (7.5, 57.1) |
| Beef or mutton (g/day) * | 0.7 (0.0, 3.3) | | | 1.0 (0.0, 4.3) | 0.5 (0.0, 5.3) | 2.0 (0.0, 10.0) |
| Poultry (g/week) * | 23.3 (6.9, 58.3) | | | 23.3 (3.8, 70.0) | 35.0 (2.9, 96.0) | 50.0 (5.8, 150.0) |
| Fish (g/week) * | 84.0 (11.7, 280.0) | | | 42.0 (3.8, 102.7) | 24.3 (0.0, 100.0) | 42.0 (2.7, 123.3) |
| Eggs (g/day) * | **8.6 (4.1, 17.1)** | | | **21.4 (13.9, 28.6)** | **50.0 (21.8, 55.5)** | **60.0 (45.7, 77.1)** |
| Dairy (g/day) * | **0.0 (0.0, 0.0)** | | | **0.0 (0.0, 28.6)** | **0.0 (0.0, 64.3)** | **107.1 (0.0, 215.0)** |
| Nuts(g/week) * | 5.8 (0.0, 28.0) | | | 2.3 (0.0, 23.3) | 2.3 (0.0, 42.0) | 4.7 (0.0, 46.7 |
| **Staple-preferred Pattern** | | |  |  |  |  |
| Rice (g/day) * | **100.0 (38.0, 180.0)** | | | **152.0 (85.7, 300.0)** | **150.0 (57.1, 300.0)** | **28.6 (6.7, 150.0)** |
| Wheat (g/day) * | **28.6 (7.1, 51.4)** | | | **50.0 (14.3, 100.0)** | **100.0 (57.1, 150.0)** | **300.0 (200.0, 375.0)** |
| Tubers (g/day) * | 14.3 (6.4, 31.0) | | | 16.7 (7.0, 38.6) | 22.9 (7.7, 47.2) | 28.6 (10.3, 57.1) |
| Legumes (g/day) * | 42.9 (15.0, 95.5) | | | 28.6 (10.0, 64.3) | 33.6 (11.9, 63.0) | 24.3 (9.0, 53.6) |
| Fresh vegetables (g/day) * | 141.9 (74.0, 266.0) | | | 154.2 (83.1, 277.1) | 216.3 (100.0, 333.6) | 162.4 (71.9, 307.0) |
| Fresh fruit (g/day) * | 38.6 (17.1, 86.2) | | | 35.2 (14.3, 73.1) | 46.3 (18.3, 91.7) | 42.9 (13.5, 95.2) |
| Pork (g/day) * | 20.0 (7.1, 42.9) | | | 24.3 (9.2, 51.4) | 28.6 (11.0, 80.0) | 21.4 (6.7, 46.3) |
| Beef or mutton (g/day) * | 2.0 (0.0, 7.1) | | | 1.1 (0.0, 5.7) | 1.3 (0.0, 5.3) | 0.0 (0.0, 3.3) |
| Poultry (g/week) * | 40.0 (9.6, 93.3) | | | 28.8 (9.3, 93.3) | 37.3 (7.0, 100.0) | 23.3 (0.0, 70.0) |
| Fish (g/week) * | 41.1 (3.8, 120.0) | | | 46.7 (9.9, 180.0) | 67.7 (3.8, 200.0) | 23.3 (0.0, 93.3) |
| Eggs (g/day) * | 20.1 (9.0, 35.7) | | | 21.4 (10.0, 47.1) | 28.6 (14.3, 57.1) | 50.0 (21.4, 60.0) |
| Dairy (g/day) * | 42.9 (0.0, 152.5) | | | 0.0 (0.0, 50.0) | 0.0 (0.0, 42.1) | 0.0 (0.0, 0.7) |
| Nuts(g/week) * | 5.8 (0.0, 60.0) | | | 4.8 (0.0, 28.0) | 6.6 (0.0, 33.5) | 0.0 (0.0, 23.3) |
| ^a^: expressed as median (P25, p75). Q1–Q4 are quartiles of each dietary pattern score. Food intakes features as the typical food of each dietary pattern are shown in bold. *: *P*<0.05, examined by Kruskal–Wallis H test. | | | | | | |

Table S3 Sociodemographics, lifestyles and health related factors across the quartiles of each dietary pattern score ^a^

| Characteristics | Meat-preferred Pattern | | | | *P* value | Plant-preferred Pattern | | | | *P* value | Eggs and Dairy Pattern | | | | *P* value | Staple-preferred Pattern | | | | *P* value |  |
| --- | --- | --- | --- | --- | --- | --- | --- | --- | --- | --- | --- | --- | --- | --- | --- | --- | --- | --- | --- | --- | --- |
|  | Q1 | Q2 | Q3 | Q4 |  | Q1 | Q2 | Q3 | Q4 |  | Q1 | Q2 | Q3 | Q4 |  | Q1 | Q2 | Q3 | Q4 |  |  |
| Age group (years)^b^ | | |  |  | <0.001 |  |  |  |  | <0.001 |  |  |  |  | 0.012 |  |  |  |  | 0.184 |  |
| 55-64 | 32.6 | 35.0 | 37.9 | 41.8 |  | 34.5 | 35.4 | 37.9 | 39.4 |  | 38.3 | 38.2 | 36.8 | 33.9 |  | 36.7 | 36.8 | 35.9 | 37.9 |  |  |
| 65-74 | 44.7 | 44.3 | 44.1 | 39.9 |  | 40.9 | 45.1 | 43.2 | 43.9 |  | 43.6 | 43.6 | 43.8 | 42.1 |  | 42.9 | 42.3 | 46.6 | 41.2 |  |  |
| ≥75 | 22.7 | 20.7 | 18.0 | 18.3 |  | 24.6 | 19.5 | 18.9 | 16.7 |  | 18.1 | 18.2 | 19.4 | 24.0 |  | 20.4 | 20.9 | 17.5 | 20.9 |  |  |
| Gender^b^ |  |  |  |  | 0.066 |  |  |  |  | 0.030 |  |  |  |  | 0.017 |  |  |  |  | <0.001 |  |
| male | 47.0 | 43.6 | 43.1 | 47.8 |  | 48.0 | 44.8 | 42.0 | 46.8 |  | 43.0 | 43.3 | 46.5 | 48.8 |  | 37.7 | 40.2 | 49.0 | 54.7 |  |  |
| female | 53.0 | 56.4 | 56.9 | 52.2 |  | 52.0 | 55.2 | 58.0 | 53.2 |  | 57.0 | 56.7 | 53.5 | 51.2 |  | 62.3 | 59.8 | 51.0 | 45.3 |  |  |
| Resident area^b^ | |  |  |  | <0.001 |  |  |  |  | <0.001 |  |  |  |  | <0.001 |  |  |  |  | <0.001 |  |
| urban | 43.7 | 47.9 | 60.0 | 46.0 |  | 34.0 | 47.4 | 57.9 | 58.4 |  | 47.9 | 46.4 | 46.2 | 57.2 |  | 67.5 | 49.2 | 51.5 | 29.5 |  |  |
| rural | 56.3 | 52.1 | 40.0 | 54.0 |  | 66.0 | 52.6 | 42.1 | 41.6 |  | 52.1 | 53.6 | 53.8 | 42.8 |  | 32.5 | 50.8 | 48.5 | 70.5 |  |  |
| Education level^b^ | | |  |  | <0.001 |  |  |  |  | <0.001 |  |  |  |  | <0.001 |  |  |  |  | <0.001 |  |
| lliteracy | 16.5 | 16.5 | 12.5 | 11.3 |  | 16.9 | 14.9 | 14.6 | 10.6 |  | 15.5 | 14.1 | 15.3 | 12.0 |  | 9.6 | 11.6 | 13.0 | 22.8 |  |  |
| ≤Primary school | 43.6 | 41.5 | 39.2 | 41.2 |  | 47.5 | 46.5 | 36.2 | 35.4 |  | 52.2 | 42.2 | 39.6 | 31.7 |  | 39.1 | 44.9 | 39.5 | 42.0 |  |  |
| ≥Secondary school | 39.9 | 42.0 | 48.2 | 47.5 |  | 35.6 | 38.6 | 49.2 | 54.0 |  | 32.3 | 43.7 | 45.1 | 56.3 |  | 51.3 | 43.5 | 47.5 | 35.2 |  |  |
| Current employment^b^ | | |  |  | <0.001 |  |  |  |  | <0.001 |  |  |  |  | <0.001 |  |  |  |  | <0.001 |  |
| yes | 21.6 | 11.7 | 11.9 | 26.5 |  | 23.4 | 19.4 | 15.1 | 13.7 |  | 20.7 | 19.6 | 18.2 | 13.2 |  | 10.0 | 14.7 | 21.7 | 25.3 |  |  |
| no | 78.4 | 88.3 | 88.1 | 73.5 |  | 76.6 | 80.6 | 84.9 | 86.3 |  | 79.3 | 80.4 | 81.8 | 86.8 |  | 90.0 | 85.3 | 78.3 | 74.7 |  |  |
| Monthly household income per capital (RMB)^b^ | | | | | <0.001 |  |  |  |  | <0.001 |  |  |  |  | <0.001 |  |  |  |  | <0.001 |  |
| <1000 | 39.2 | 25.8 | 15.6 | 13.8 |  | 30.3 | 27.6 | 21.4 | 15.0 |  | 23.7 | 21.9 | 29.5 | 19.2 |  | 15.9 | 18.1 | 24.1 | 36.3 |  |  |
| 1000-3999 | 53.0 | 66.8 | 68.3 | 58.2 |  | 56.0 | 61.2 | 64.8 | 64.4 |  | 57.5 | 64.9 | 59.7 | 64.3 |  | 66.8 | 65.5 | 58.4 | 55.5 |  |  |
| ≥4000 | 7.8 | 7.4 | 16.1 | 28.0 |  | 13.7 | 11.2 | 13.8 | 20.6 |  | 18.8 | 13.2 | 10.8 | 16.5 |  | 17.3 | 16.4 | 17.4 | 8.2 |  |  |
| Physical activity level^b^ | | | |  | 0.001 |  |  |  |  | <0.001 |  |  |  |  | 0.208 |  |  |  |  | <0.001 |  |
| low | 32.1 | 33.9 | 34.3 | 32.5 |  | 39.4 | 31.2 | 30.6 | 31.7 |  | 35.8 | 32.6 | 31.4 | 33.2 |  | 33.9 | 34.5 | 29.6 | 34.9 |  |  |
| medium | 30.6 | 36.2 | 35.3 | 31.7 |  | 29.4 | 33.7 | 36.3 | 34.4 |  | 32.2 | 32.0 | 34.6 | 35.0 |  | 36.6 | 35.3 | 32.4 | 29.6 |  |  |
| high | 37.3 | 29.9 | 30.4 | 35.8 |  | 31.2 | 35.1 | 33.1 | 33.9 |  | 32.0 | 35.4 | 34.0 | 31.8 |  | 29.5 | 30.2 | 38.0 | 35.5 |  |  |
| Smoking^b^ | |  |  |  | 0.028 |  |  |  |  | 0.034 |  |  |  |  | 0.957 |  |  |  |  | <0.001 |  |
| ever/current | 24.9 | 22.7 | 21.8 | 26.8 |  | 25.0 | 23.2 | 21.4 | 26.6 |  | 23.5 | 24.3 | 23.9 | 24.4 |  | 18.3 | 19.6 | 24.8 | 33.5 |  |  |
| never | 75.1 | 77.3 | 78.2 | 73.2 |  | 75.0 | 76.8 | 78.6 | 73.4 |  | 76.5 | 75.7 | 76.1 | 75.6 |  | 81.7 | 80.4 | 75.2 | 66.5 |  |  |
| Alcohol intake^b^ | |  |  |  | 0.004 |  |  |  |  | <0.001 |  |  |  |  | 0.021 |  |  |  |  | 0.001 |  |
| ever/current | 16.1 | 15.8 | 16.2 | 20.9 |  | 13.9 | 15.3 | 16.4 | 23.3 |  | 17.6 | 15.5 | 15.9 | 20.1 |  | 15.0 | 15.0 | 18.6 | 20.3 |  |  |
| never | 83.9 | 84.2 | 83.8 | 79.1 |  | 86.1 | 84.7 | 83.6 | 76.7 |  | 82.4 | 84.5 | 84.1 | 79.9 |  | 85.0 | 85.0 | 81.4 | 79.7 |  |  |
| Meeting sleep duration recommendation^b^ | | | | | 0.091 |  |  |  |  | 0.004 |  |  |  |  | 0.508 |  |  |  |  | 0.116 |  |
| yes | 62.5 | 64.2 | 66.7 | 67.0 |  | 61.6 | 63.6 | 68.3 | 66.9 |  | 63.7 | 66.3 | 64.3 | 66.0 |  | 65.9 | 66.8 | 65.5 | 62.1 |  |  |
| no | 37.5 | 35.8 | 33.3 | 33.0 |  | 38.4 | 36.4 | 31.7 | 33.1 |  | 36.3 | 33.7 | 35.7 | 34.0 |  | 34.1 | 33.2 | 34.5 | 37.9 |  |  |
| Medical history^b^ | | |  |  | <0.001 |  |  |  |  | 0.138 |  |  |  |  | <0.001 |  |  |  |  | <0.001 |  |
| yes | 40.3 | 37.0 | 36.1 | 31.7 |  | 36.6 | 33.8 | 36.0 | 38.6 |  | 30.9 | 34.8 | 37.5 | 41.8 |  | 30.6 | 36.9 | 36.2 | 41.3 |  |  |
| no | 59.7 | 63.0 | 63.9 | 68.3 |  | 63.4 | 66.2 | 64.0 | 61.4 |  | 69.1 | 65.2 | 62.5 | 58.2 |  | 69.4 | 63.1 | 63.8 | 58.7 |  |  |
| Obesity^b^ | | |  |  | <0.001 |  |  |  |  | 0.115 |  |  |  |  | 0.005 |  |  |  |  | 0.173 |  |
| yes | 15.5 | 14.9 | 13.6 | 8.7 |  | 11.6 | 13.0 | 15.1 | 13.0 |  | 10.4 | 13.1 | 15.6 | 13.7 |  | 13.2 | 11.5 | 13.3 | 14.8 |  |  |
| no | 84.5 | 85.1 | 86.4 | 91.3 |  | 88.4 | 87.0 | 84.9 | 87.0 |  | 89.6 | 86.9 | 84.4 | 86.3 |  | 86.8 | 88.5 | 86.7 | 85.2 |  |  |
| Central obesity^b^ | | |  |  | <0.001 |  |  |  |  | 0.001 |  |  |  |  | <0.001 |  |  |  |  | 0.135 |  |
| yes | 52.4 | 50.5 | 44.8 | 38.4 |  | 42.5 | 45.4 | 50.7 | 47.5 |  | 39.0 | 48.3 | 50.1 | 48.7 |  | 48.2 | 43.7 | 46.2 | 48.0 |  |  |
| no | 47.6 | 49.5 | 55.2 | 61.6 |  | 57.5 | 54.6 | 49.3 | 52.6 |  | 61.0 | 51.7 | 49.9 | 51.3 |  | 51.8 | 56.3 | 53.8 | 52.0 |  |  |
| Energy (kcal)^c^ | 1505.4±619.6 | 1490.2±582.3 | 1392.5±569.3 | 1699.9±667.4 | <0.001 | 1530.8±608.2 | 1457.4±564.2 | 1380.6±524.2 | 1719.2±718.5 | <0.001 | 1572.2±624.5 | 1437.2±569.5 | 1430.2±554.6 | 1648.4±697.3 | <0.001 | 1571.9±627.5 | 1447.6±603.3 | 1395.2±558.7 | 1673.3±652.1 | <0.001 |  |
| ^a^: Q1–Q4 are quartiles of each dietary pattern score.  ^b^: Values are expressed as % and examined using chi-square test. ^c^: Values are expressed as mean±SD and examined using Kruskal–Wallis H test. | | | | | | | | | | | | | | | | | | | | | |
